# Adipocyte-specific expression of C-type natriuretic peptide suppresses lipid metabolism and adipocyte hypertrophy in adipose tissues in mice fed high-fat diet

**DOI:** 10.1038/s41598-018-20469-z

**Published:** 2018-02-01

**Authors:** Cho-Rong Bae, Jun Hino, Hiroshi Hosoda, Cheol Son, Hisashi Makino, Takeshi Tokudome, Tsutomu Tomita, Kiminori Hosoda, Mikiya Miyazato, Kenji Kangawa

**Affiliations:** 1 Department of Biochemistry, National Cerebral and Cardiovascular Center Research Institute, Suita, Osaka Japan; 2 Departments of Regenerative Medicine and Tissue Engineering, National Cerebral and Cardiovascular Center Research Institute, Suita, Osaka Japan; 3 Division of Endocrinology and Metabolism, National Cerebral and Cardiovascular Center, Suita, Osaka Japan; 4 Omics Research Center and National Cerebral and Cardiovascular Center, Suita, Osaka Japan; 5 Biobank, National Cerebral and Cardiovascular Center, Suita, Osaka Japan

## Abstract

C-type natriuretic peptide (CNP) is expressed in diverse tissues, including adipose and endothelium, and exerts its effects by binding to and activating its receptor, guanylyl cyclase B. Natriuretic peptides regulate intracellular cGMP and phosphorylated vasodilator-stimulated phosphoprotein (VASP). We recently revealed that overexpression of CNP in endothelial cells protects against high-fat diet (HFD)-induced obesity in mice. Given that endothelial CNP affects adipose tissue during obesity, CNP in adipocytes might directly regulate adipocyte function during obesity. Therefore, to elucidate the effect of CNP in adipocytes, we assessed 3T3-L1 adipocytes and transgenic (Tg) mice that overexpressed CNP specifically in adipocytes (A-CNP). We found that CNP activates the cGMP–VASP pathway in 3T3-L1 adipocytes. Compared with Wt mice, A-CNP Tg mice showed decreases in fat weight and adipocyte hypertrophy and increases in fatty acid β-oxidation, lipolysis-related gene expression, and energy expenditure during HFD-induced obesity. These effects led to decreased levels of the macrophage marker F4/80 in the mesenteric fat pad and reduced inflammation. Furthermore, A-CNP Tg mice showed improved glucose tolerance and insulin sensitivity, which were associated with enhanced insulin-stimulated Akt phosphorylation. Our results suggest that CNP overexpression in adipocytes protects against adipocyte hypertrophy, excess lipid metabolism, inflammation, and decreased insulin sensitivity during HFD-induced obesity.

## Introduction

The growing public-health problem of obesity is characterized by the excessive accumulation of adipose tissue due to an imbalance between energy intake and energy expenditure^1^. Adipose tissue plays a critical role in regulating energy balance and lipid metabolism^2^. In addition, white adipose tissue (WAT) stores excessive energy as triglycerides and secretes adipokines that mediate lipid metabolism, secretion of inflammatory cytokines, and insulin sensitivity^3^.

Natriuretic peptides (NPs), which include atrial NP (ANP), brain NP (BNP), and C-type NP (CNP), are associated with obesity, insulin resistance, and metabolic syndrome^4–7^. In particular, C-type NP (CNP) is an endogenous peptide that binds its receptor guanylyl cyclase B (GCB)^8,9^, thus inducing cGMP production^10^. cGMP regulates the cardiovascular system and adipose tissue function^11,12^. In addition, the accumulation of intracellular cGMP activates its downstream target vasodilator-stimulated phosphoprotein (VASP)^13^. CNP is locally produced and acts as an autocrine–paracrine regulator^14^ that is expressed in a wide variety of tissues, including brain, lung, heart, kidney, bone, and endothelium. We recently revealed that CNP and GCB are expressed in adipose tissue^7^. Transgenic (Tg) mice with endothelial cell-specific overexpression of CNP are protected against visceral adipose tissue hypertrophy, systemic inflammation, and insulin resistance during the development of obesity due to feeding of a high-fat diet (HFD)^7^. However, direct the effects of CNP on adipocytes are unclear.

In the current study, we found that treatment with CNP stimulates the cGMP–VASP pathway in 3T3-L1 adipocytes. Furthermore, we placed the *CNP* gene under the control of the adiponectin promoter^15^ and used this construct to generate Tg mice that selectively overexpressed CNP in adipocytes (A-CNP). The results revealed the CNP–GCB–cGMP–VASP pathway in adipocytes and showed that the overexpression of CNP in adipocytes decreased adipocyte hypertrophy in WAT and ameliorated metabolic disorders during HFD-induced obesity in mice.

## Results

### CNP activates the cGMP–VASP pathway in 3T3-L1 adipocytes

**Figure 1:**
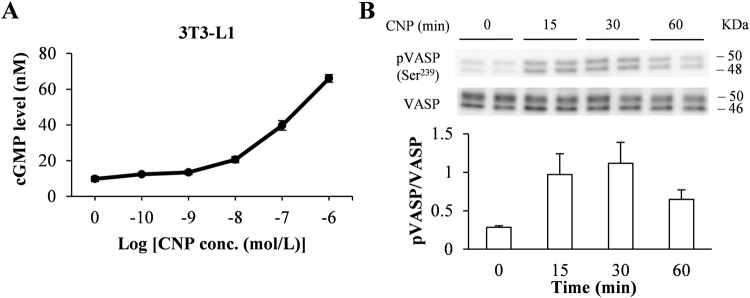
C-type natriuretic peptide (CNP) upregulated cGMP level and phosphorylation of vasodilator-stimulated phosphoprotein (VASP) in 3T3-L1 adipocytes. (**A**) We exposed 3T3-L1 adipocytes to the indicated concentrations of CNP for 30 min, then lysed the cells and measured the cGMP concentrations of the lysates. (**B**) The levels of phosphorylated VASP (pVASP) after exposure of 3T3-L1 adipocytes to CNP (1 × 10^−7^ M) were assessed by using western blot analysis. The data are presented as means ± SEM. *n* = 5 (A); *n* = 4 (B).

To determine whether CNP is a regulator in adipocytes, we measured cGMP production in response to CNP treatment. CNP dose-dependently increased the intracellular cGMP levels of 3T3-L1 adipocytes and isolated mouse mature adipocytes (Fig. 1A and Supplementary Figure S1). We then measured the levels of VASP phosphorylation after CNP treatment in 3T3-L1 adipocytes. The CNP-induced increase of the pVASP (Ser^239^) content in 3T3-L1 cells peaked at 30 min and then decreased (Fig. 1B). This result indicates that CNP–GCB-dependent cGMP production induced the phosphorylation of VASP in 3T3-L1 adipocytes.

### *CNP* mRNA expression is upregulated in the adipose tissue of A-CNP Tg mice fed HFD

**Figure 2:**
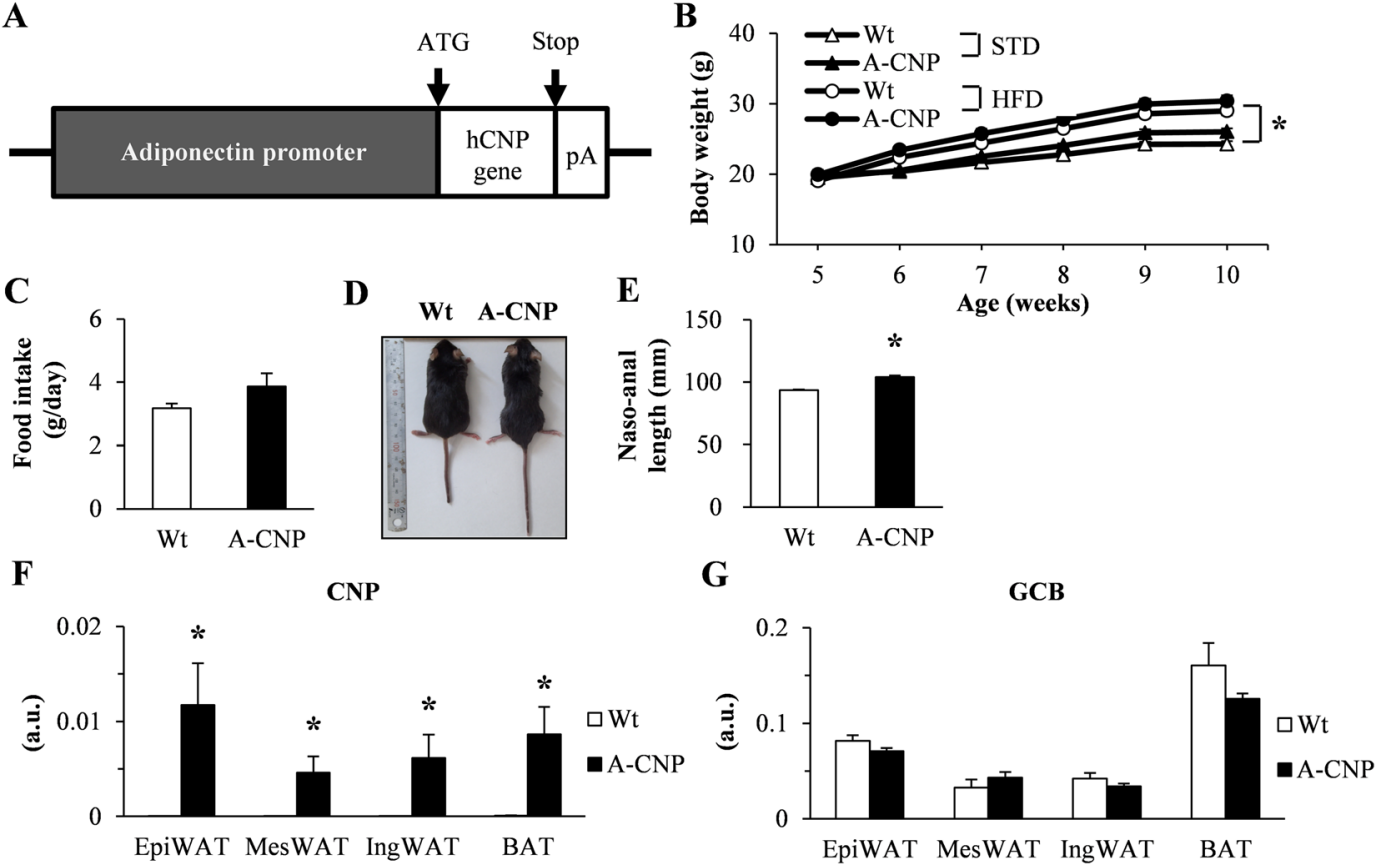
Characteristics of wild-type (Wt) and A-CNP transgenic (Tg) mice fed a high-fat diet (HFD). (**A**) Schematic representation of the A-CNP Tg transgene, (**B**) body weight, (**C**) food intake, (**D**) macroscopic appearance, and (**E**) naso-anal length of Wt and A-CNP Tg mice on HFD. (**F**) CNP and (**G**) guanylyl cyclase B (GCB) mRNA expression in adipose tissue (epididymal, mesenteric, and inguinal white adipose tissue [EpiWAT, MesWAT, and IngWAT, respectively] and brown adipose tissue [BAT]) from Wt and A-CNP Tg mice fed HFD at 10 weeks. pA, polyA tail. a.u., arbitrary units (copy number of gene of interest/copy number of reference gene [ribosomal protein 36B4]). The data are presented as means ± SEM. *n* = 16–17 (**B**,**C** and **E**); *n* = 10 (**F** and **G**); (**B**) ^*^P < 0.05 for standard (STD)-fed group vs HFD-fed group; (**E** and **F**) ^*^P < 0.05.

To investigate the effects of CNP in adipocytes, we generated Tg mice that overexpress CNP under the control of the adipocyte-specific adiponectin promoter (A-CNP) (Fig. 2A). Intake of HFD is associated with increased adipose tissue^16^. In addition, our previous results indicated that *CNP* mRNA expression in adipose tissues differs between HFD-fed mice and those fed a standard diet (STD)^7^. Therefore, in the current study, we focused on CNP overexpression in the adipose tissue of mice on HFD. Body weight was increased in wild-type (Wt) and A-CNP Tg mice on HFD compared with STD, but body weight did not differ between Wt and A-CNP Tg mice in either the STD- or HFD-fed groups from 5 to 10 weeks of age (Fig. 2B). Food intake did not differ between HFD-fed Wt and A-CNP Tg mice (Fig. 2C). However, naso–anal length was significantly greater in A-CNP Tg compared with Wt mice (Fig. 2D and E), likely because CNP promotes bone growth, given that endothelial cell-specific CNP Tg mice demonstrated increased naso–anal length also^7,17^.

Under HFD conditions, A-CNP Tg mice showed significantly increased *CNP* mRNA expression in various adipose depots, including epididymal white adipose tissue (EpiWAT), mesenteric adipose tissue (MesWAT), inguinal white adipose tissue (IngWAT), and brown adipose tissue (BAT), compared with Wt mice (Fig. 2F). However, *GCB* mRNA expression in adipose tissues did not differ between groups (Fig. 2G).

We then examined *CNP* and *GCB* mRNA expression in stromal vascular fraction (SVF) and mature adipocytes from the visceral adipose tissue (EpiWAT and MesWAT) of HFD-fed mice. The *CNP* mRNA level in the mature adipocytes of EpiWAT (Supplementary Figure S2A) and MesWAT (Supplementary Figure S2B) of A-CNP Tg mice was significantly higher than in Wt mice. In contrast, *GCB* mRNA expression showed no difference between groups in SVF as well as mature adipocytes of EpiWAT (Supplementary Figure S2C) and MesWAT (Supplementary Figure S2D). In addition, the concentration of CNP was significantly higher in the plasma and adipose tissues (BAT, IngWAT, and MesWAT) of A-CNP Tg compared with Wt mice (Table 1). In contrast, the plasma ANP and BNP concentration were similar between the groups (Supplementary Table S2).

**Table 1:** CNP concentration in plasma and tissues of A-CNP Tg mice.

	Wt	A-CNP
Plasma (pg/mL)	N.D.	1.30 ± 0.19
Brown adipose tissue (pg/mg)	N.D.	0.39 ± 0.03
Inguinal white adipose tissue (pg/mg)	N.D.	0.20 ± 0.02
Mesenteric white adipose tissue (pg/mg)	N.D.	0.16 ± 0.14

Plasma CNP concentration was analyzed (CNP-22 RIA kit, Phoenix Pharmaceuticals, Burlingame, CA, USA) in mice fed standard diet; all other concentrations were measured in mice fed the high-fat diet. N. D, not detectable. The data are presented as means ± SEM. *n* = 3–4.

### The increased fatty acid oxidation in adipocytes regulates the WAT weight of A-CNP Tg mice during HFD-induced obesity

**Figure 3:**
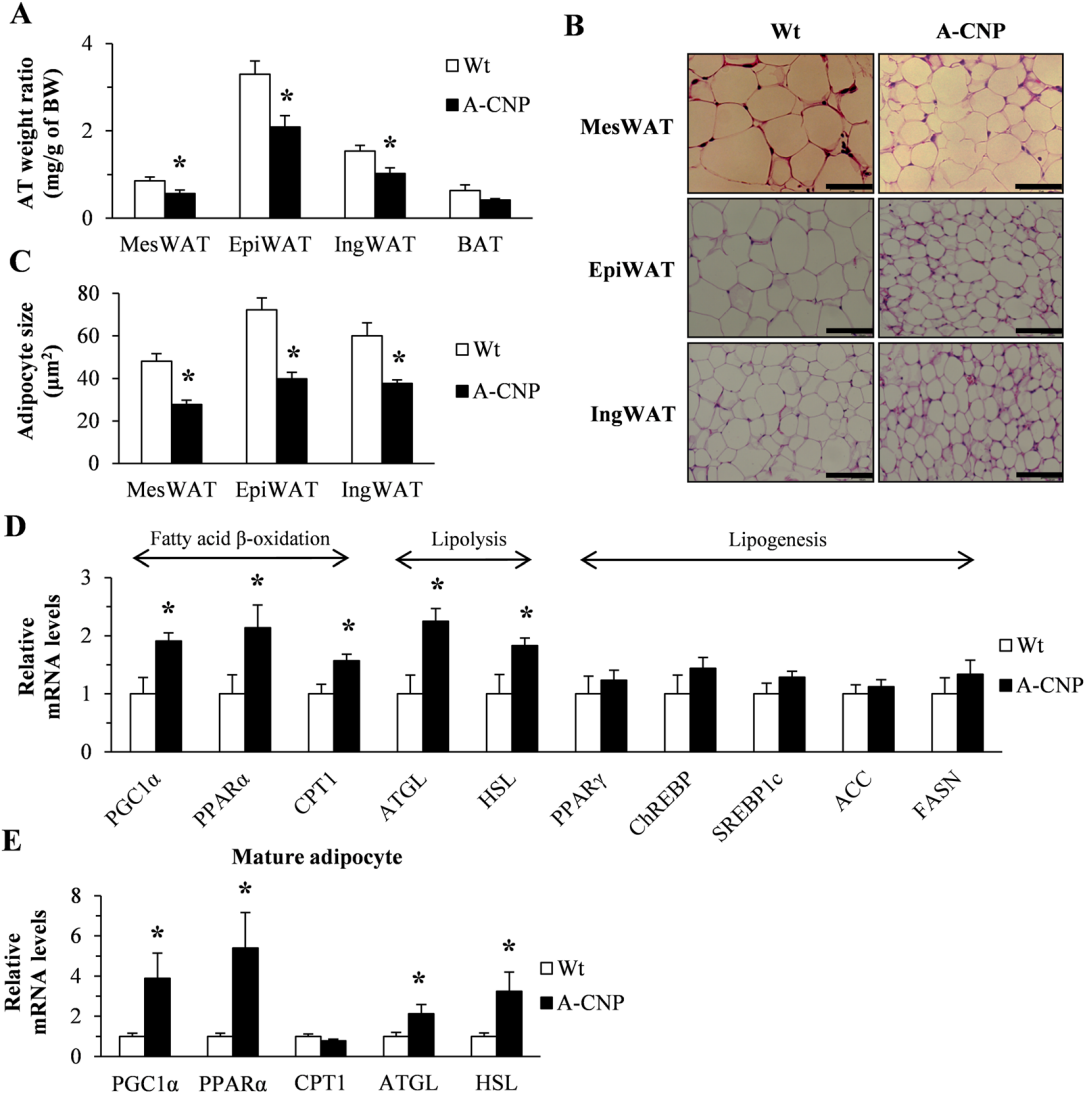
A-CNP Tg mice had decreased WAT weight and increased expression of fatty acid oxidation–related genes during HFD-induced obesity. (**A**) Adipose depot weight normalized to body weight. (**B** and **C**) Adipocyte size in MesWAT, EpiWAT, and IngWAT. (**D**) Quantitative polymerase chain reaction (qPCR) analysis of mRNA expression in MesWAT of genes involved in β-oxidation, lipolysis, and lipogenesis. (**E**) qPCR analysis of mRNA expression in mature adipocytes of genes involved in β-oxidation and lipolysis. Scale bars, 50 μm. The data are presented as means ± SEM. *n* = 15–16 (**A**); *n* = 5 (**B**); *n* = 10 (**C**); n = 8–10 (**D**); *n* = 4–5 (**E**); ^*^P < 0.05.

Compared with Wt mice fed HFD, A-CNP Tg mice had significantly lower WAT:body weight ratio; adipocyte size in MesWAT, EpiWAT, and IngWAT (Fig. 3A–C); triglyceride content of MesWAT; and serum levels of total cholesterol and free fatty acids (Table 2). In EpiWAT and IngWAT, the expression of several genes involved in lipid metabolism— particularly fatty acid β-oxidation, lipolysis, and lipogenesis—did not differ between Wt and A-CNP Tg mice (Supplementary Figure S3A and B). In contrast, in MesWAT, the expression of genes associated with fatty acid β-oxidation and lipolysis, such as peroxisome proliferator-activated receptor γ coactivator-1α (*PGC1α*), peroxisome proliferator-activated receptor α (*PPARα*), carnitine palmitoyltransferase 1 (*CPT1*), adipose triglyceride lipase (*ATGL*), and hormone-sensitive lipase (*HSL*) was significantly greater in A-CNP Tg mice compared with Wt mice, but the lipogenesis-related genes peroxisome proliferator-activated receptor γ (*PPARγ*), carbohydrate responsive element binding protein (*ChREB*P), sterol regulatory element-binding protein (*SREBP1c*), acetyl-CoA carboxylase (*ACC*), and fatty acid synthase (*FAS*N) were expressed similarly between groups (Fig. 3D). We therefore focused on the MesWAT of A-CNP Tg mice during HFD-induced obesity.

**Table 2:** Adipose tissue lipid and serum parameters in wild-type (Wt) and A-CNP Tg mice fed high-fat diet (HFD).

	Wt	A-CN**P**
***Mesenteric adipose tissue***		
Triglycerides (mg/g)	6.6 ± 0.9	3.9 ± 0.4*
***Serum***		
Triglycerides (mg/dL)	160.4 ± 17.1	135.2 ± 26.5
Total cholesterol (mg/dL)	156.2 ± 5.1	128.7 ± 11.0*
Free fatty acids (mEq/L)	0.7 ± 0.04	0.5 ± 0.04*
Insulin (ng/mL)	0.7 ± 0.20	0.4 ± 0.03
Leptin (ng/mL)	5.2 ± 0.70	4.2 ± 1.20
Adiponectin (ng/mL)	2.9 ± 0.20	2.7 ± 0.20

The data are presented as means ± SEM. *n* = 9–10. ^*^P < 0.05 between Wt and A-CNP mice.

We then examined the expression of genes related to fatty acid β-oxidation and lipolysis in mature MesWAT adipocytes. In these adipocytes, mRNA levels of *PGC1α*, *PPARα*, *ATGL*, and *HSL* were significantly higher in A-CNP Tg mice than in Wt mice (Fig. 3E). These results suggest that the decrease in the MesWAT weight of A-CNP Tg mice might be due to changes in the expression of genes related to fatty acid β-oxidation and lipolysis in MesWAT adipocytes.

### A-CNP Tg mice fed HFD show increases in energy expenditure and thermogenesis markers in IngWAT

**Figure 4:**
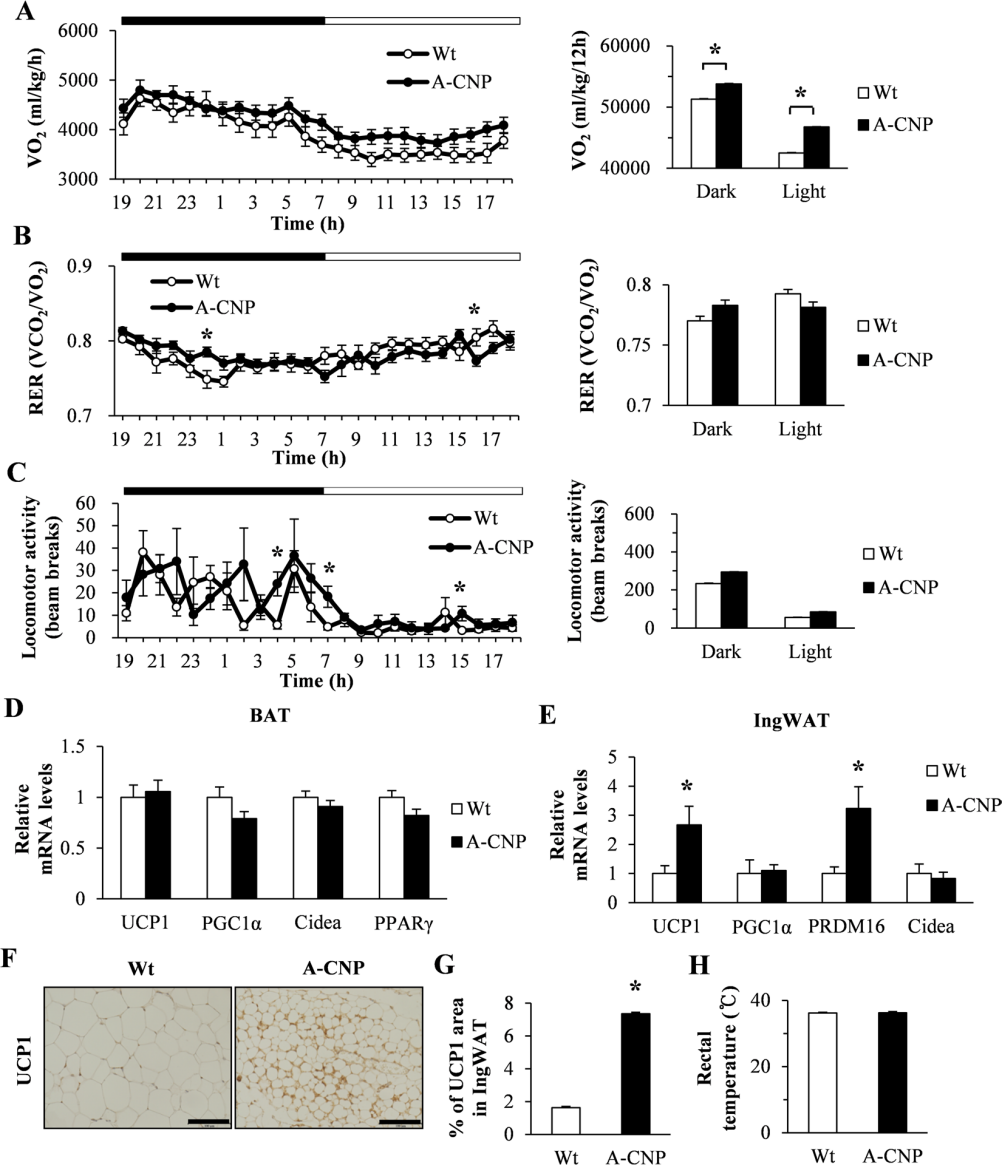
Energy metabolism of A-CNP Tg mice during HFD-induced obesity. (**A**) Oxygen consumption (VO_2_), (**B**) Respiratory exchange ratio (RER), and (**C**) locomotor activity in Wt and A-CNP Tg mice. qPCR expression analysis of thermogenesis-related genes in (**D**) BAT and (**E**) IngWAT. (**F** and **G**) Immunohistochemical analysis and quantification of UCP1 in the IngWAT of Wt and A-CNP Tg mice. (**H**) Rectal temperature in Wt and A-CNP Tg mice. Data are presented as means ± SEM. *n* = 8 (**A**–**C**); *n* = 10–12 (**D** and **E**); *n* = 2 (**F** and **G**); *n* = 7 (**H**); ^*^P < 0.05.

We further investigated the metabolic effect of CNP overexpression in the adipose tissue of A-CNP Tg mice. In the HFD-fed mice, oxygen consumption (VO_2_) was significantly increased during both phases of the light:dark cycle in A-CNP Tg mice, but their respiratory exchange ratio (RER) and locomotor activity were similar to those of Wt mice (Fig. 4A–C). Because BAT has emerged as an important player in energy metabolism^18^, we analyzed the expression of thermogenesis-related genes in the BAT of our HFD-fed mice. However, mRNA transcript levels of uncoupling protein 1 (*UCP1*), *PGC1α*, cell death–inducing DNA fragmentation factor α-like effector A (*Cidea*), and *PPARγ* did not differ between A-CNP Tg and Wt mice (Fig. 4D). Recent studies have described the “beiging” of WAT, where the expression levels of transcriptional regulators of BAT function are induced^19^. Therefore, we examined key transcriptional regulators of BAT development, including UCP1, PGC1α, positive regulatory domain-containing 16 (PRDM16), and Cidea. The mRNA expression of *UCP1* and *PRDM16* was significantly higher in the IngWAT of A-CNP Tg compared with that of Wt mice (Fig. 4E). In addition, UCP1 protein was more abundant in the IngWAT of A-CNP Tg than in that of Wt mice (Fig. 4F and G). However, body temperature, blood pressure, and heart rate were similar between groups (Fig. 4H and Supplementary Table S3). These data suggest that the “beiging” of their IngWAT contributes to the increased energy expenditure of A-CNP Tg mice.

### Inflammation in MesWAT is decreased during HFD-induced obesity in A-CNP Tg mice

**Figure 5:**
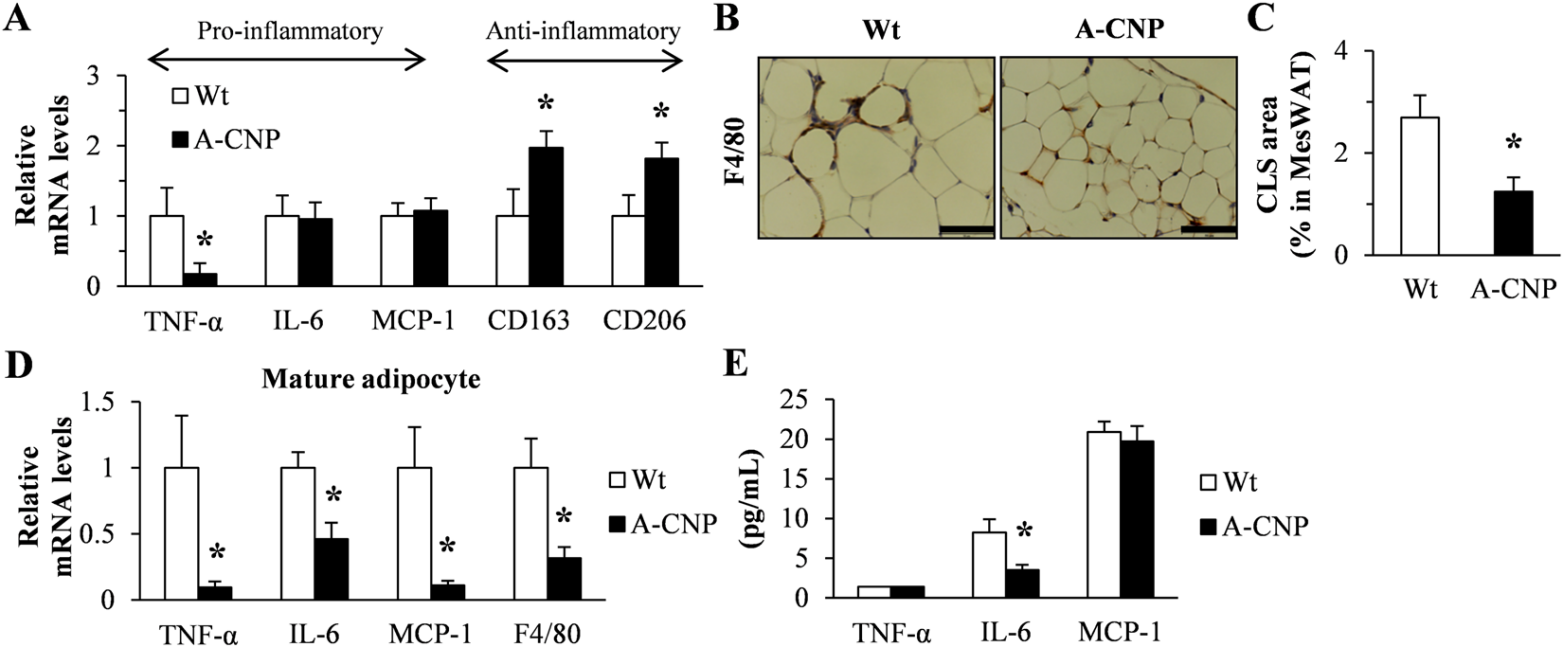
A-CNP Tg mice showed protection against inflammation during HFD-induced obesity. (**A**) Expression of inflammatory cytokines in MesWAT was determined by qPCR analysis. (**B** and **C**) Immunohistochemical analysis and quantification of crown-like structures in A-CNP Tg mice by using anti-F4/80 staining. Scale bars, 50 μm. (**D**) Expression of inflammatory cytokines in mature adipocytes of MesWAT was determined by qPCR analysis. (**E**) Serum inflammatory cytokine levels were measured by ELISA. The data are presented as means ± SEM. *n* = 10 (**A**); *n* = 5 (**B** and **C**); *n* = 4 (**D**); *n* = 10 (**E**); ^*^P < 0.05.

Obesity is associated with chronic low-grade systemic inflammation in adipose tissue; both of these conditions contribute to increased circulating levels of pro-inflammatory cytokines^20^. Therefore, we evaluated the potential anti-inflammatory role of CNP expression in adipose tissue. First, MesWAT from A-CNP Tg mice showed significantly decreased mRNA expression of the pro-inflammatory marker tumour necrosis factor-α (*TNF-α*), with concomitant significant increases in mRNA levels of the anti-inflammatory M2 macrophage markers clusters of differentiation 163 (*CD163*) and *CD206* (Fig. 5A). Consistent with this finding, the proportion of F4/80-positive crown-like structures (CLSs) in adipocytes was lower in A-CNP Tg than in Wt mice (Fig. 5B and C). In other words, A-CNP Tg mice had fewer macrophage clusters for forming CLSs. Because the infiltration of macrophages into adipose tissue contributes to the increased expression of inflammatory cytokines during obesity^21^, we assessed the levels of various inflammatory markers in mature adipocytes isolated from MesWAT. Compared with Wt mice, A-CNP Tg mice had decreased gene expression of *TNF-α*, interleukin-6 (*IL-6*), monocyte chemoattractant protein-1 (*MCP-1*), and *F4/80* in mature adipocytes of MesWAT (Fig. 5D) but decreased serum levels of IL-6 (Fig. 5E).

### A-CNP Tg mice have improved insulin sensitivity during HFD-induced obesity

**Figure 6:**
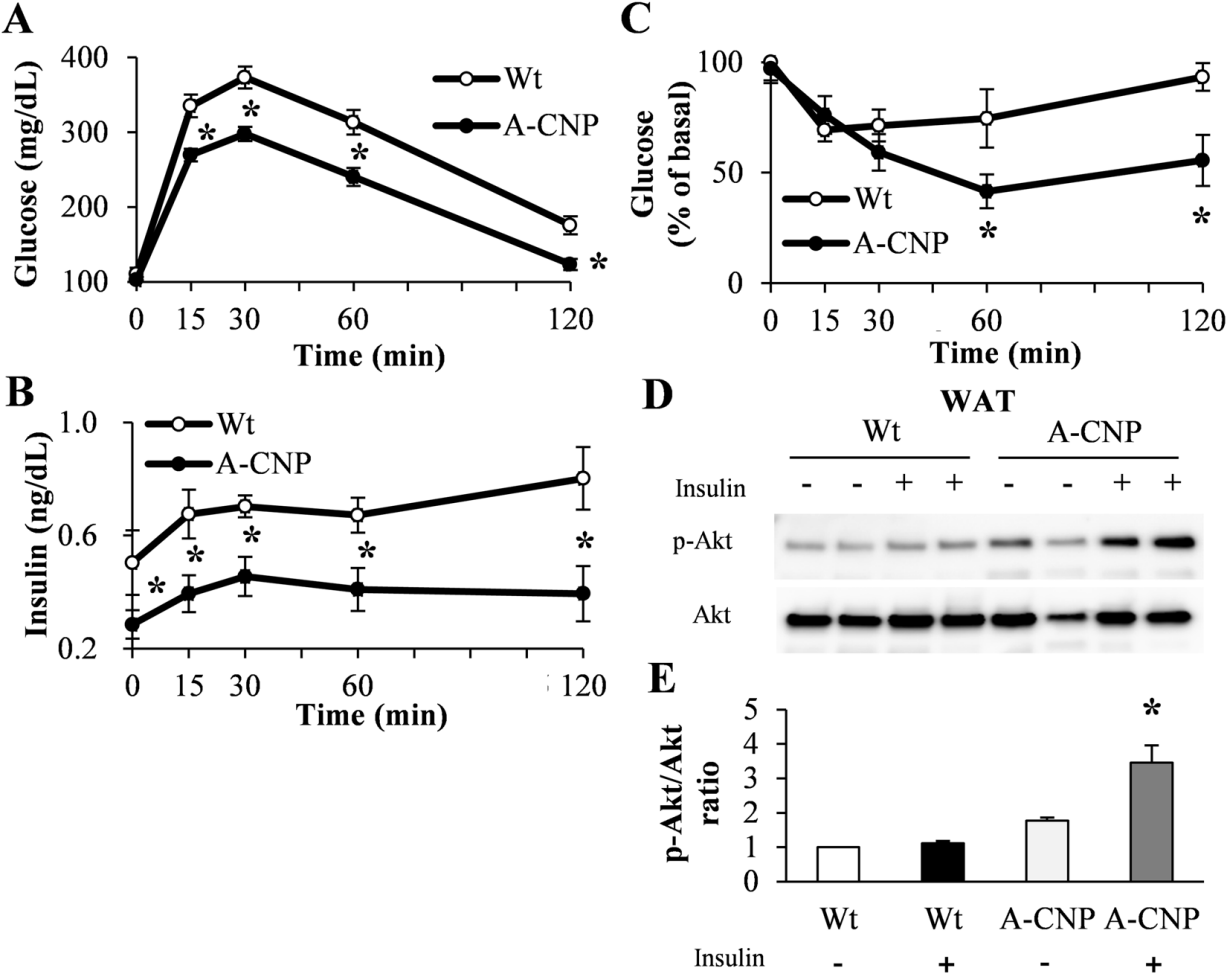
A-CNP Tg mice have increased insulin sensitivity during HFD-induced obesity. Results of (**A**) glucose tolerance test (GTT), (**B**) serum insulin levels during GTT, and (**C**) insulin tolerance test (ITT) in HFD-fed mice at 10 weeks. (**D** and **E**) Akt activation in MesWAT after insulin injection (1 U/kg IP, 8 min). (**D**) Western blot analysis of MesWAT extracts showing phospho- (Ser^473^) Akt (p-Akt) levels under control (PBS, indicated as [−]) and insulin-treated (+) conditions. (**E**) Densitometric quantitation of the pAkt:Akt ratio. *n* = 12–14 (**A** and **C**); *n* = 4 (**D and**  **E**); ^*^P < 0.05; (**E**), *P* < 0.05 vs Wt mice without insulin injection (−).

Adipose tissues regulate systemic glucose metabolism and insulin sensitivity^22^. In HFD-fed mice, glucose and insulin levels during glucose tolerance test (GTT) were lower in A-CNP Tg mice than in Wt mice (Fig. 6A and B), as was the glucose level during insulin tolerance test (ITT) (Fig. 6C). In addition, insulin-stimulated Akt phosphorylation, an indicator of insulin signaling^23^, was augmented in the MesWAT of A-CNP Tg mice compared with Wt mice (Fig. 6D and E). These findings prompted us to use immunohistochemistry to analyze the morphology and proliferation of pancreatic β-cells. Insulin- and Ki67-positive areas in the pancreatic islets were significantly smaller in HFD-fed A-CNP Tg than in Wt mice (Supplementary Figure S4).

## Discussion

In the current study, we showed that the CNP–GCB–cGMP–VASP pathway is active in adipocytes and that overexpression of CNP in adipocytes suppresses adipocyte hypertrophy in fat pads, reduces fat weight, enhances energy expenditure, decreases inflammation, and improves insulin sensitivity in HFD-fed Tg mice. Our previous study indicated that *CNP* mRNA levels in adipose tissue are increased in mice fed HFD compared with STD and that systemic endothelial cell-specific CNP overexpression in Tg mice decreases fat weight, systemic inflammation, and insulin resistance^7^. Together, these results suggest that the effects of CNP in adipose tissues counteract with HFD-induced obesity.

Both ANP and BNP induce a cGMP-dependent pathway and VASP phosphorylation at Ser239 in adipocytes^24,25^. Our current results indicate that exogenous CNP dose-dependently increases intracellular cGMP levels in 3T3-L1 and mature adipocytes; this finding is consistent with the CNP-induced cGMP increases in lung fibroblasts and rhabdomyosarcoma cells^26,27^. In addition, CNP increased the phosphorylation of VASP in 3T3-L1 adipocytes. Enhancing NP in adipose tissue regulates diet-induced obesity and insulin resistance^28^. Furthermore, the cGMP–VASP pathway decreases inflammation and insulin resistance in various peripheral organs including adipose tissue, liver, and vasculature^29–31^. Therefore, we surmise that the CNP–GCB–cGMP–VASP pathway in adipocytes modulates inflammation and insulin resistance.

In our study, body weight and food intake were similar between Wt and A-CNP Tg mice fed HFD, however the decreased WAT (MesWAT, EpiWAT, and IngWAT) weight of A-CNP Tg mice was reflected as decreased adipocyte hypertrophy and triglyceride content in MesWAT. In addition, A-CNP Tg mice showed changes in lipid metabolism due to increased expression of fatty acid β-oxidation and lipolysis-related genes in MesWAT and mature adipocytes. By controlling the lipolysis of adipose tissue, ANP and BNP contribute to the regulation of lipid metabolism^32–36^. In addition, the ANP/BNP–cGMP pathway has direct effects on mitochondrial biogenesis in skeletal muscle^28,37^. Therefore, we believe that the CNP–GCB–cGMP–VASP pathway in adipocytes modulates fatty acid β-oxidation and lipolysis in the adipose tissue of A-CNP Tg mice fed HFD.

We showed that A-CNP Tg mice had higher energy expenditure, as indicated by their increased oxygen consumption than Wt mice. These results suggest that their increased energy expenditure contributes to their decreased WAT weight. The increased oxygen consumption is due to thermogenesis in BAT and “beiging” of WAT^38–40^. Furthermore, the development of beiging adipocytes within WAT has potential anti-obesity and insulin-sensitizing effects^41^. We recently demonstrated that endothelial cell-specific overexpression of CNP in Tg mice increased oxygen consumption through the increased expression of thermogenesis-related genes, including *UCP1*, *Cidea*, *PRDM16*, and *PPARγ* in BAT^7^. Likewise, in the current study, WAT from A-CNP Tg mice had increased expression of thermogenesis-related genes during HFD conditions. Therefore, the results of our previous and current studies show that CNP in peripheral tissues increases energy expenditure through increased beiging of WAT and activation of BAT; these effects depend on the overexpression of CNP specifically in adipocytes or endothelial cells.

Adipocyte hypertrophy is associated with inflammatory infiltration^42^. Compared with Wt mice, our A-CNP Tg mice had decreased adipocyte hypertrophy and lipid accumulation in visceral adipose tissue, especially in MesWAT. Visceral adipose tissue is associated with an increased clinical risk of metabolic and cardiovascular diseases^43^. However, enhanced cGMP signaling inhibits inflammation in visceral adipose tissues during obesity^7,29,44^. All markers of inflammation that we evaluated were decreased in the mature adipocytes of A-CNP Tg mice, whereas some, but not all, markers were decreased in the MesWAT and serum. In addition, the livers of A-CNP Tg mice showed partial suppression of the pro-inflammatory state characteristic of HFD-induced obesity (Supplementary Figure S5). Several other studies similarly reported that CNP exerts anti-inflammatory effects in various cells and tissues, including lung, chondrocytes, and cartilage^26,45–50^. Inflammation in adipose tissue is primarily mediated by macrophages^51^, whereas adipocytes are sensitive to the effects of TNF-α^52^. Adipocyte hypertrophy and chronic inflammation in adipose tissue profoundly contribute to the development of obesity and obesity-associated metabolic dysfunctions, such as insulin resistance^53,54^. In the current study, compared with Wt mice, A-CNP Tg mice had lower glucose levels during GTT and ITT and greater insulin-stimulated Akt phosphorylation. Consequently, given that CNP in adipocytes is an important regulator of metabolism, this improvement in insulin sensitivity may reflect not only decreased fat accumulation but also decreased concentrations of pro-inflammatory cytokines.

In conclusion, CNP activated the GCB –cGMP–VASP pathway in adipocytes. Furthermore, CNP overexpression in adipocytes decreased adipocyte hypertrophy in adipose tissue, increased fatty acid β-oxidation and lipolysis in visceral MesWAT, increased energy expenditure, decreased inflammation, and improved insulin sensitivity. Therefore, adipocyte-specific expression of CNP may protect against the development of the metabolic disease associated with obesity.

## Materials and Methods

### CNP and 3T3-L1 cell culture

CNP was donated by Asubio Pharma Co., Ltd. (Kobe, Japan). 3T3-L1 cells were purchased from American Type Culture Collection (Manassas, VA, USA) and cultured in Dulbecco’s modified Eagle medium (DMEM) containing 10% calf serum and 100 U/mL penicillin–streptomycin (Invitrogen, Carlsbad, CA, USA).

### cGMP assay in 3T3-L1 cells and primary mature adipocytes

3T3-L1 cells (1 × 10^6^ cells/well) and primary mature adipocytes (8 × 10^5^ cells/well) were cultured for 30 min in 1 mM 3-isobutyl-1-methylxanthine, 0.05 M hydroxyethyl–piperazineethane–sulfonic acid (HEPES) buffer (pH 7.4), 0.1% bovine serum albumin (Sigma–Aldrich, St. Louis, MO, USA) with or without CNP (1 × 10^−10^ to 1 × 10^−6^ M). The cGMP content of the cells was measured by using cGMP kit (Cisbio, Tokyo, Japan) according to the manufacturer’s instructions.

### Animals

C57BL/6 J mice were obtained from CLEA Japan (Tokyo, Japan). All experiments were approved by the Animal Care and Use Committee of the National Cerebral and Cardiovascular Center Research Institute (Osaka, Japan), whose laboratory animal facilities comply with the “Basic Policies for the Conduct of Animal Experimentation in the Ministry of Health, Labour, and Welfare” according to assessment by the Center for Accreditation of Laboratory Animal Care and Use, Japan Health Sciences Foundation, and were performed in accordance with the approved guidelines. All mice were housed under a 12:12-h light:dark cycle and had unrestricted access to STD (12 kcal% fat, 29 kcal% protein, and 59 kcal% carbohydrate; CE-2, CLEA Japan) or HFD (57 kcal% fat, 20 kcal% protein, and 23 kcal% carbohydrate; High Fat Diet 32, CLEA Japan) and water. For the HFD-induced obesity model, mice were fed a HFD beginning at 5 weeks. All mice were weighed once weekly. Body length was measured as the nose-to-anus length at necropsy. Unless otherwise stated, mice were fasted for 16 hours before being euthanized.

### Generation of A-CNP Tg mice

The A**-**CNP Tg construct was generated by using the Red/ET Counter Selection Bacterial Artificial Chromosome (BAC) Modification Kit (Gene Bridges, Heidelberg, Germany). A human BAC containing the CNP gene (clone RP11-46M10, Thermo Fisher Scientific/Invitrogen, Waltham, MA, USA) and a mouse BAC clone containing the adiponectin gene promoter (clone RP23-364K13, Thermo Fisher Scientific/Invitrogen) were used to construct the transgene. The Tg construct was purified and microinjected into the pronucleus of C57BL/6 J mouse embryos by using standard techniques. Tg F1 mice were identified by Southern blot analysis and then were mated with C57BL/6 J mice to expand the population of A-CNP Tg mice. All experiments involving A-CNP Tg mice used male Tg mice and their sex-matched Wt littermates.

### Isolation of SVF and primary mature adipocytes

MesWAT was fractionated as described previously^55^, with some modifications. Briefly, fat pads were isolated from 10-week-old Wt and A-CNP Tg mice. A maximum of 1 g of tissue was digested with 20 mg of collagenase type VIII (Sigma–Aldrich) in Krebs–Ringer bicarbonate HEPES buffer containing 1% bovine serum albumin (Sigma–Aldrich) at 37 °C for 1 h. After centrifugation, primary mature adipocytes were obtained from the upper layer, and the SVF was obtained from the precipitated cells.

### Biochemical analysis of serum and tissues

Serum triglyceride, total cholesterol, and free fatty acid concentrations were measured by using commercial kits (Wako, Osaka, Japan). The triglyceride and total cholesterol of tissues such as MesWAT and liver were extracted with a chloroform:methanol solution (2:1, vol/vol) by using the Bligh and Dyer method^56^. Briefly, the chloroform:methanol solution was added into the homogenized tissue, vortexed, and centrifuged; the lower phase was collected and evaporated at room temperature under a fume hood. The remaining semi-dried pellets were dissolved in 1% Triton X-100 (Nacalai Tesque, Kyoto, Japan). The triglyceride and total cholesterol content of tissues were analyzed by using the same enzymatic kits as for the serum analyses. The serum concentrations of insulin, leptin, and adiponectin were determined by using ELISA kits (insulin and leptin: Morinaga, Yokohama, Japan; adiponectin: Otsuka Pharmaceutical, Tokyo, Japan). Serum concentrations of TNF-α, IL-6, and MCP-1 were determined by using Quantikine ELISA kits (R&D Systems, Minneapolis, MN, USA).

### Metabolic assessment

The VO_2_, RER, and locomotor activity of HFD-fed Wt and A-CNP Tg mice were assessed in a metabolic monitoring system (CLAMS, Columbus Instruments, Columbus, OH, USA) for 1weeks. The RER was calculated as the ratio between the VCO_2_ and VO_2_. The VO_2_, VCO_2_, RER, and locomotor activity were monitored at 8- to 14-min intervals. Mice were placed in individual metabolic cages with free access to water and food. Animal were maintained at a controlled temperature of 24 ± 1 °C and humidity of 50–54%. After the mice had adapted to the environment of the metabolic cages at least 1~2 days, and data collection occurred on days devoid of any husbandry-associated events, such as cage cleaning and diet supply. Body temperature was measured by inserting a sensor (measurement range, 22–42 °C; measurement error, ±0.1 °C; ATB-1100, Nihon Kohden, Tokyo, Japan) into the rectum.

### Histology and immunohistochemical analysis

Samples of adipose tissue, pancreas, and liver were fixed in 4% paraformaldehyde in phosphate buffer solution (Wako) for 24 h, embedded in paraffin, sectioned at 4 μm, and stained with hematoxylin and eosin. For immunohistochemistry, paraffin-embedded sections were stained with monoclonal anti-UCP1 (dilution, 1:200; Abcam, Cambridge, MA, USA), anti-insulin (Histofine, Nichirei, Tokyo, Japan), anti-Ki67 (dilution, 1:1000; Abcam, Cambridge, MA, USA), and anti-F4/80 (AbD Serotec, Oxford, UK) antibodies. Images were acquired by using an FSX100 system (Olympus, Tokyo, Japan), and the UCP1 and F4/80 areas were evaluated by using cellSens Dimension software version 1.6 (Olympus). Histologic images were analyzed to calculate the sizes of adipocytes and insulin- and Ki67-positive areas by using Image J software (National Institutes of Health, Bethesda, MD, USA). For the analysis, 5 random images were captured for every sample.

### RNA isolation and quantitative RT-PCR analysis

Total RNA from tissues was isolated using TRIzol Reagent (Invitrogen). First-strand cDNA was synthesized from total RNA by using a commercially available kit (QuantiTect Reverse Transcription kit, Qiagen, Hamburg, Germany). Quantitative real-time PCR analysis was performed using the SYBR Premix Ex Taq (Takara, Shiga, Japan) and a LightCycler 480 System II (Roche Applied Science, Indianapolis, IN, USA). The sequences of the gene-specific primers used are given in Supplementary Table S1. Gene copy numbers were derived from a standard curve generated by using serially diluted plasmid DNA and were normalized against the mRNA level of ribosomal protein 36B4.

### Western blot analysis

Cells were lysed with RIPA buffer (1% NP-40 buffer, 0.1% SDS, 1% sodium deoxycholate, 20 mM Tris-HCl [pH 7.4], 150 mM Nacl, 5 mM EDTA) containing phosphatase inhibitor cocktails (#04080-11 and #07574-61, Nacalai Tesque, Kyoto, Japan). Tissues were lysed in NP-40 buffer (1% NP-40, 20 mM Tris-HCl [pH 7.4], 150 mM Nacl, 5 mM EDTA) supplemented with protease and phosphatase inhibitor cocktails. The concentration of total protein was determined by using the Pierce 660 nm Protein Assay Reagent (Thermo Fisher Scientific). Proteins were separated by 4% to 15% SDS-PAGE (Bio-Rad, Hercules, CA, USA) and transferred to a polyvinylidene fluoride membrane (Millipore, Billerica, MA, USA). The membrane was incubated in polyvinylidene difluoride blocking reagent (Toyobo, Osaka, Japan) at room temperature for 20 min and was then incubated at 4 °C overnight with the appropriate primary antibody diluted in Can Get Signal Solution 1 (Toyobo). The primary antibodies used for the analysis were anti-phospho-VASP (Ser^239^) (#3114, Cell Signaling Technology, Danvers, MA, USA), anti-VASP (#3132, Cell Signaling), anti-phospho-Akt (Ser^473^) (#9271, Cell Signaling), and anti-Akt (#9272, Cell Signaling). An image of the membrane was obtained by using a LAS-4000 mini luminescent image analyzer (GE Healthcare UK, Little Chalfont, England), and band intensities were quantitated by using Multi Gauge software (version 3.11, GE Healthcare UK).

### Glucose and insulin tolerance tests

Mice were fasted overnight (~16 h) before intraperitoneal administration of the glucose challenge dose (1 g/kg body weight); blood glucose levels were measured at the indicated time points before and after glucose challenge. For ITT, mice were fasted for 4 h prior to intraperitoneal injection of insulin (0.75 U/kg body weight; Eli Lilly and Company, Indianapolis IN, USA). Blood samples were drawn from the tail vein for measurements of blood glucose by using a glucometer (Sanwa Kagaku Kenkyusho, Nagoya, Japan).

### Measurement of ANP, BNP, and CNP concentration in plasma and tissues

Plasma samples were collected with EDTA-2Na (2 mg/ml) and aprotinin (500 kIU/ml). Tissue samples were boiled for 5 min to inactivate intrinsic proteases, acidified with acetic acid, and homogenized^57^. The plasma and tissue samples were treated with Sep-Pak C18 cartridges (Waters, Milford, MA, USA). The plasma concentrations of ANP and BNP in mice were measured with each radioimmunoassay (RIA) system, we developed, using polyclonal rabbit antiserum raised against alpha-mouse ANP [7–23] and mouse pro-BNP [68–95], respectively. The CNP concentration in plasma and tissues were determined by using a CNP RIA kit (Phoenix Pharmaceuticals, Belmont, CA, USA).

### Measurement of blood pressure and heart rate

Blood pressure and heart rate were measured in conscious animals using the tail-cuff method (Softron, BP-98A, Tokyo, Japan).

### Statistical analysis

All values are expressed as means ± SEM. Data were analyzed by using SPSS software (version 12.0 for Windows, IBM, Armonk, NY, USA). Statistical significance was evaluated by using Student’s *t*-test and one-way ANOVA with *post hoc* Tukey–Kramer testing. Differences were considered significant at *P* < 0.05.

## Electronic supplementary material

Supplementary information
