# Supplementary material for: Adipocyte-specific expression of C-type natriuretic peptide suppresses lipid metabolism and adipocyte hypertrophy in adipose tissues in mice fed high-fat diet
Source: Sci Rep. 2018 Feb 1;8:2093. doi: 10.1038/s41598-018-20469-z (PMC5794866; doi:10.1038/s41598-018-20469-z)
Supplement: Supplementary file 1 — Supplementary information [file 41598_2018_20469_MOESM1_ESM.pdf]

SUPPLEMENTARY INFORMATION

Adipocyte-specific expression of C-type natriuretic peptide suppresses lipid metabolism and adipocyte hypertrophy in adipose tissues in mice fed high-fat diet

Cho-Rong Bae[1], Jun Hino[1], Hiroshi Hosoda[2], Cheol Son[3, 4], Hisashi Makino[3], Takeshi Tokudome[1], Tsutomu Tomita[3, 5], Kiminori Hosoda[3], Mikiya Miyazato[1], and Kenji Kangawa[1]

[1]Department of Biochemistry, [2]Departments of Regenerative Medicine and Tissue Engineering, National Cerebral and Cardiovascular Center Research Institute, 5-7-1, Fujishirodai, Suita, Osaka, Japan; [3]Division of Endocrinology and Metabolism, [4]Omics Research Center, and [5]Biobank, National Cerebral and Cardiovascular Center, Suita, Osaka, Japan.

Supplementary Tables and legends

Supplementary Table S1. Primers used for real-time qPCR analysis of gene expression

Gene	Forward primer (5′ to 3′)	Reverse primer (5′ to 3′)
ACC	CCCATCCAAACAGAGGGAAC	CTGACAAGGTGGCGTGAAG
ATGL	TGACCATCTGCCTTCCAGA	TGTAGGTGGCGCAAGACA
CD163	TCTCAGTGCCTCTGCTGTCA	CGCCAGTCTCAGTTCCTTCT
CD206	CCACAGCATTGAGGAGTTTG	ACAGCTCATCATTTGGCTCA
ChREBP	GGCCTGGCTGGAACAGTA	CGAAGGGAATTCAGGACAGT
CNP	ACCGAAGGTCCCGAGAACCCC	GACTTGGTGTCCACACGCAGGTCC
CPT1	TGCACTACGGAGTCCTGCAA	GGACAACCTCCATGGCTCAG
F4/80	CTTTGGCTATGGGCTTCCAGTC	GCAAGGAGGGCAGAGTTGATCGTG
FASN	GCTGCTGTTGGAAGTCAGC	AGTGTTCGTTCCTCGGAGTG
GCB	GTCGCTGCGGGGATCCAGTTACG	ATGTTGGGAGGGTCTATGCAGGC
HSL	GCGCTGGAGGAGTGTTTTT	CCGCTCTCCAGTTGAACC
IL-6	CCAGTTGCCTTCTTGGGACTGATG	GTAATTAAGCCTCCGACTTGTGTGAA
MCP-1	GCAGGTGTCCCAAAGAAGCTGTAG	CAGAAGTGCTTGAGGTGGTTGTGG
PGC1α	CCCTGCCATTGTTAAGACC	TGCTGCTGTTCCTGTTTTC
PPARα	CTGAGACCCTCGGGGAAC	AAACGTCAGTTCACAGGGAAG
PPARγ	AGGCCGAGAAGGAGAAGCTGTTG	TGGCCACCTCTTTGCTCTGCTC
SREBP1c	TTCCTCAGACTGTAGGCAAATCT	AGCCTCAGTTTACCCACTCCT
TNF-α	TGGCCCAGACCCTCACACTCAGATC	GCCTTGTCCCTTGAAGAGAACCTGG
36B4	TCATTGTGGGAGCAGACAATGTGG	AGGTCCTCCTTGGTGAACACAAAG

ACC, acetyl CoA carboxylase; ATGL, adipose triglyceride lipase; CD163, cluster of differentiation 163; CD206, cluster of differentiation 206; ChREBP, carbohydrate-responsive element-binding protein; CNP, C-type natriuretic peptide; CPT1, carnitine palmitoyltransferase 1; FASN, fatty acid synthase; GCB, guanylyl cyclase B; HSL, hormone-sensitive lipase; IL-6, interleukin-6; MCP-1, monocyte chemoattractant protein-1; PGC1α, peroxisome proliferator-activated receptor gamma coactivator 1α; PPARα, peroxisome proliferator-activated receptor α; PPARγ, peroxisome proliferator-activated receptor γ; SREBP1c, sterol regulatory element-binding protein 1c; TNF-α, tumor necrosis factor-α.

Supplementary Table S2. Plasma concentrations of ANP and BNP in mice

	**Wt**	**A-CNP**
ANP (fmol/mL)	85.03 ± 10.45	55.48 ± 1.10
BNP (fmol/mL)	53.18 ± 6.52	49.62 ± 1.59

The data are presented as means ± SEM. n = 3.

Supplementary Table S3. **Blood pressure in Wt and A-CNP Tg mice**

	Blood pressure (mm Hg)			Heart rate (bpm)
	Systolic	Mean	Diastolic	
Wt	103.1 ± 2.3	76.9 ± 2.8	64.0 ± 3.1	673 ± 21
A-CNP	102.9 ± 2.6	71.7 ± 4.0	56.4 ± 5.0	640 ± 33

The data are presented as means ± SEM. *n* = 4–5.

Supplementary Figures and legends

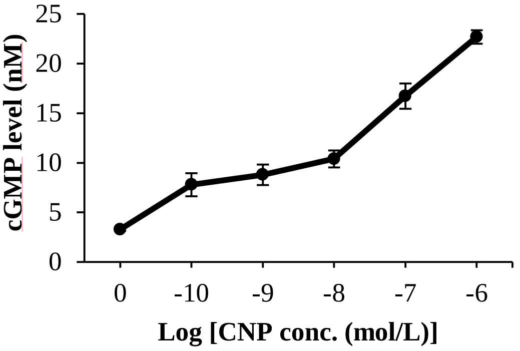

Supplementary Figure S1. CNP increases cGMP level in mature adipocytes isolated from mouse adipose tissue. The data are presented as means ± SEM. *n* = 4.

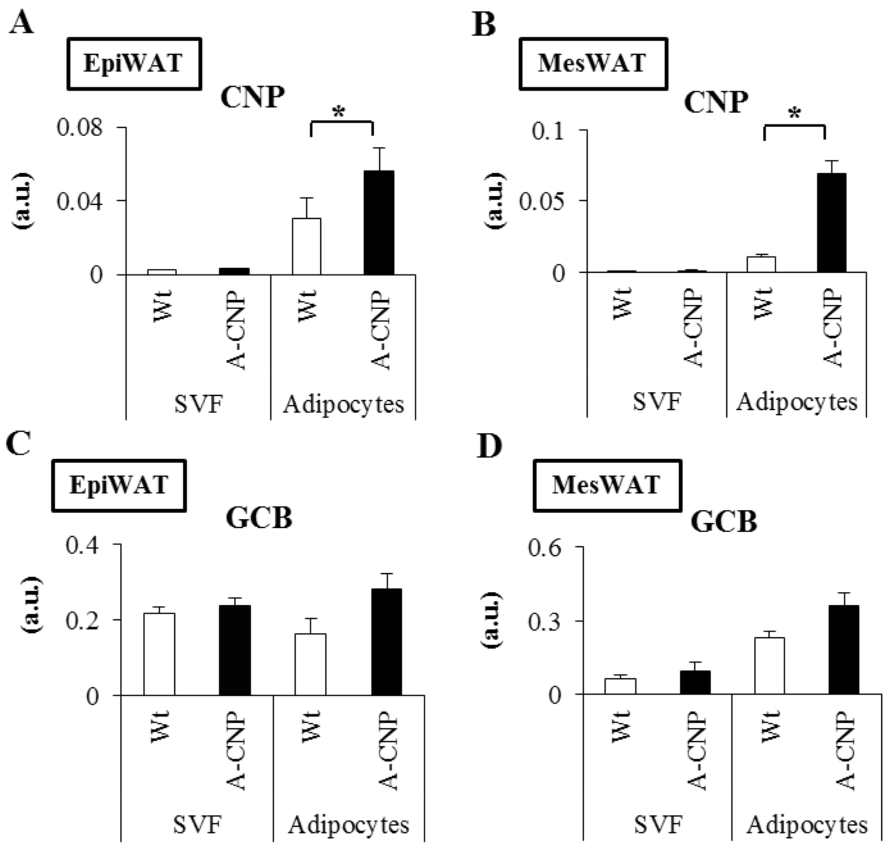

Supplementary Figure S2. CNP and GCB mRNA expression in the SVF and mature adipocytes of the visceral WAT of Wt and A-CNP Tg mice with HFD-induced obesity. (A-D) CNP and GCB mRNA expression in the stromal vascular fraction (SVF) and mature adipocytes of the epididymal white adipose tissue (EpiWAT) and mesenteric white adipose tissue (MesWAT) of Wt and Ad-CNP Tg mice fed HFD. a.u., arbitrary units (copy number of gene of interest / copy number of reference gene [ribosomal protein 36B4]). a.u., arbitrary units. The data are presented as means ± SEM. n = 5 (A–D); *****, $P < 0.05$.

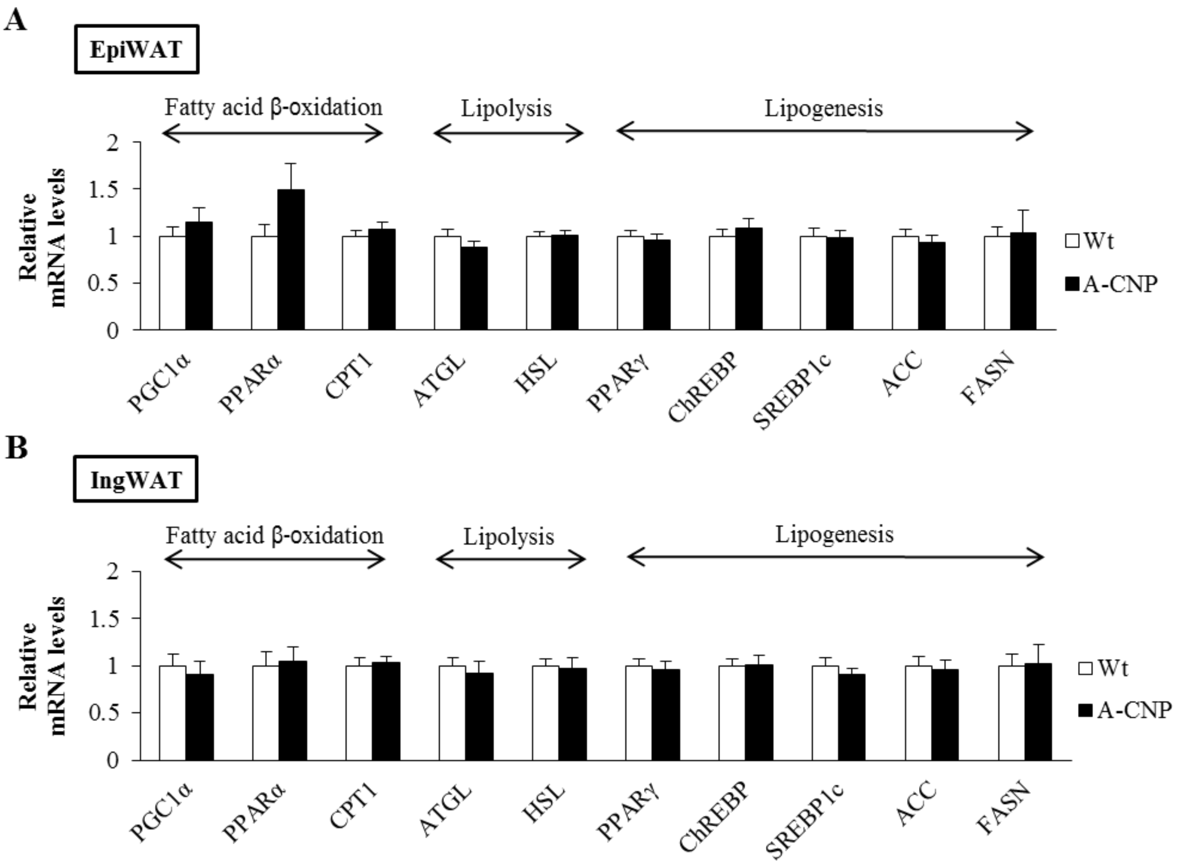

Supplementary Figure S3. Lipid metabolism related genes in EpiWAT and IngWAT of of Wt and A-CNP Tg mice with HFD-induced obesity. qPCR analysis of mRNA expression of genes involved in (A) fatty acid β-oxidation, lipolysis, and lipogenesis in epididymal white adipose tissue (EpiWAT) and inguinal white adipose tissue (IngWAT). The data are presented as means ± SEM. *n* = 8–10 (A and B).

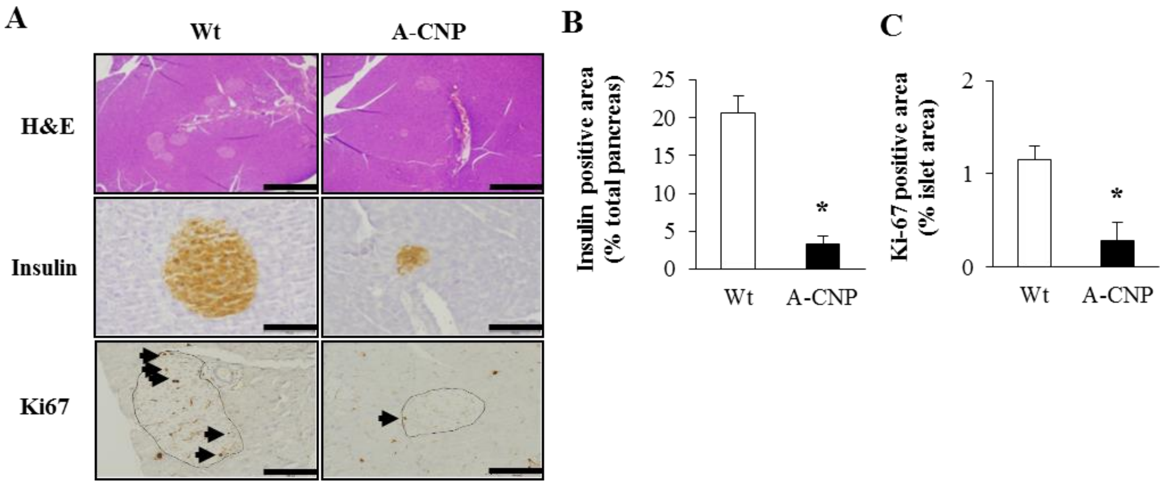

Supplementary Figure S4. Beta cell proliferation in Wt and A-CNP Tg mice with HFD-induced obesity. (A) Histology (hematoxylin and eosin [H&E] stain) and immunohistochemical quantification of the (B) insulin- (C) and Ki67-positive areas in pancreatic tissue from Wt and A-CNP Tg mice. Scale bars: H&E, 500 μm; insulin and Ki67, 100 μm. The data are presented as means ± SEM. $n = 5$ (A); $n = 9$ (B and C); *****, $P < 0.05$.

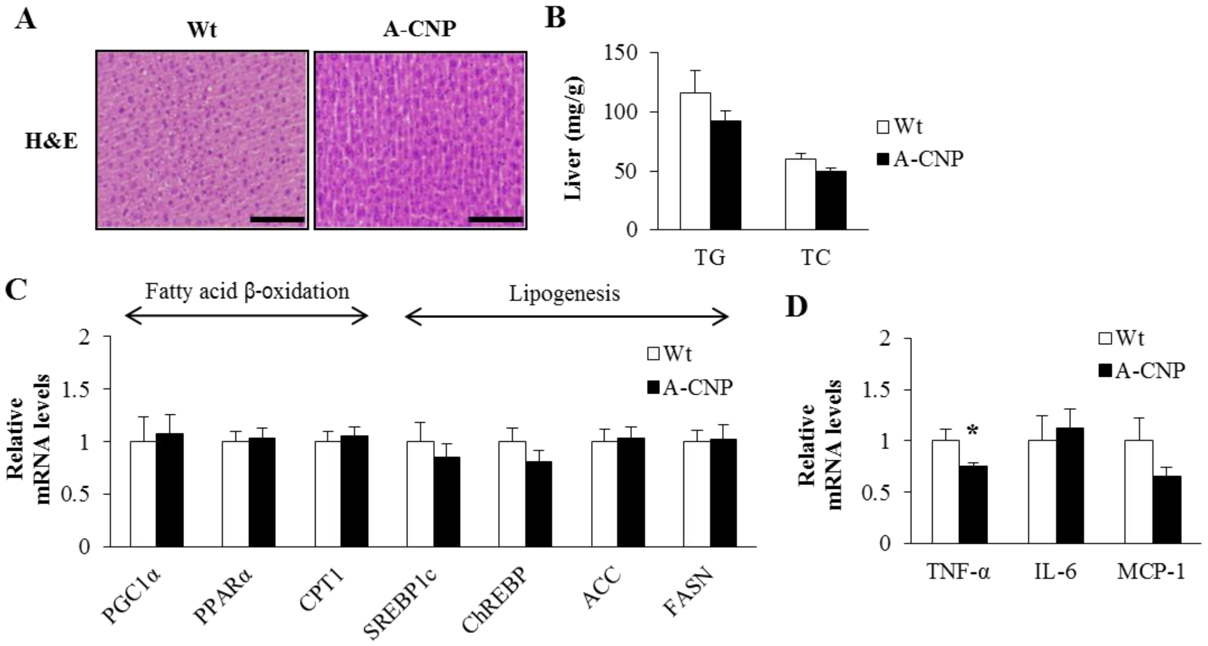

Supplementary Figure S5. Characterization of liver in Wt and A-CNP Tg mice with HFD-induced obesity. (A) Histology of liver sections stained with hematoxylin and eosin (H&E). (B) Triglyceride (TG) and total cholesterol (TC) content. Expression levels of genes involved in (C) fatty acid β-oxidation and lipogenesis, and (D) pro-inflammatory state were measured using qPCR analysis. Scale bars: 50 μm. The data are presented as means ± SEM. n = 5 (A); n = 8 (B); n = 9–11 (C and D); *****, $P < 0.05$.

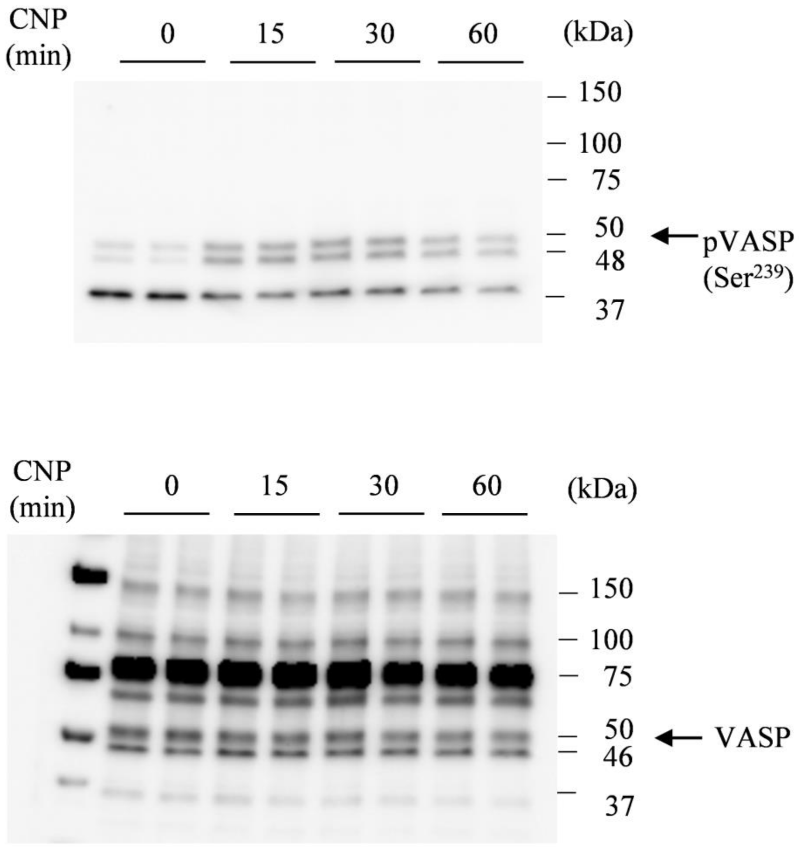

Supplementary Figure S6. The full-length blots are display of cropped blots from the main Figure 1B.
